# Factors regulating the coastal nutrient filter in the Baltic Sea

**DOI:** 10.1007/s13280-019-01282-y

**Published:** 2019-11-09

**Authors:** Jacob Carstensen, Daniel J. Conley, Elin Almroth-Rosell, Eero Asmala, Erik Bonsdorff, Vivi Fleming-Lehtinen, Bo G. Gustafsson, Camilla Gustafsson, Anna-Stiina Heiskanen, Urzsula Janas, Alf Norkko, Caroline Slomp, Anna Villnäs, Maren Voss, Mindaugas Zilius

**Affiliations:** 1 Department of Bioscience, Aarhus University, Frederiksborgvej 399, 4000 Roskilde, Denmark; 2 Department of Geology, Lund University, Sölvegatan 12, 223 62 Lund, Sweden; 3 SMHI, Sven Källfelts gata 15, 426 71 Västra Frölunda, Sweden; 4 Tvärminne Zoological Station, University of Helsinki, J.A. Palmenin tie 260, 10900 Hanko, Finland; 5 Environmental and Marine Biology, Åbo Akademi University, BioCity, 20500 Turku, Finland; 6 Finnish Environment Institute, Latokartanonkaari 11, 00790 Helsinki, Finland; 7 Stockholm University Baltic Sea Centre, 106 91 Stockholm, Sweden; 8 Department of Experimental Ecology of Marine Organisms, Institute of Oceanography, University of Gdańsk, al. Marsz. J. Pilsudskiego 46, 81-378 Gdynia, Poland; 9 Department of Earth Sciences, Utrecht University, Princetonlaan 8A, 3584 CB Utrecht, The Netherlands; 10 Department of Biological Oceanography, Leibniz Institute of Baltic Sea Research, Seestr. 15, 18119 Rostock, Germany; 11 Marine Research Institute, Universiteto al. 17, 92294 Klaipeda, Lithuania

**Keywords:** Biogeochemistry, Climate change, Coastal filter, Eutrophication, Hypoxia, Nutrient management

## Abstract

The coastal zone of the Baltic Sea is diverse with strong regional differences in the physico-chemical setting. This diversity is also reflected in the importance of different biogeochemical processes altering nutrient and organic matter fluxes on the passage from land to sea. This review investigates the most important processes for removal of nutrients and organic matter, and the factors that regulate the efficiency of the coastal filter. Nitrogen removal through denitrification is high in lagoons receiving large inputs of nitrate and organic matter. Phosphorus burial is high in archipelagos with substantial sedimentation, but the stability of different burial forms varies across the Baltic Sea. Organic matter processes are tightly linked to the nitrogen and phosphorus cycles. Moreover, these processes are strongly modulated depending on composition of vegetation and fauna. Managing coastal ecosystems to improve the effectiveness of the coastal filter can reduce eutrophication in the open Baltic Sea.

## Introduction

The open Baltic Sea is one of the most studied marine systems in the world, with a profound scientific basis for integrated management of the sea and its watershed (Reusch et al. 2018). This includes managing nutrient inputs from land and the atmosphere to mitigate the most prominent regional problem, eutrophication. Although the adverse effects of eutrophication in the open Baltic Sea have received most attention (Carstensen et al. 2014a; Kahru and Elmgren 2016), coastal eutrophication prevails around large parts of the Baltic Sea, manifested by massive algal blooms, loss of benthic vegetation and fauna as well as spreading hypoxia (Bonsdorff et al. 1997; Conley et al. 2011).

Nutrient and organic matter inputs from land enter the Baltic Sea through a broad variety of coastal ecosystems, including lagoons, archipelagos, river-dominated estuaries, embayments and open coastal stretches, and these coastal systems transform, retain and remove these substances through different biogeochemical processes with rates spanning several orders of magnitude across the Baltic Sea coastal zone (Asmala et al. 2017). Importantly, sediments play a key role in the retention and permanent removal of nutrients and organic matter. However, most studies have focused on processes in deep dark and muddy sediments, whereas less is known about the role of shallower sandy sediments, with or without sufficient light to support benthic primary producers, despite the fact that such habitats are prevalent in many coastal ecosystems (McGlathery et al. 2007). The heterogeneity of sediment types and the inhabiting biota are dictated mainly by the complex coastal bathymetry, which implies large spatial variability within coastal ecosystems. In addition, temporal variability of biogeochemical processes should in general be substantially larger in the coastal zone compared to the open Baltic Sea, due to the larger sensitivity to variable inputs from land and changing physico-chemical conditions. Consequently, more measurements are needed to constrain transformation, retention and removal processes in the coastal zone due to the enormous variability in space and time.

The attenuation of material fluxes from land to sea is often termed the “coastal filter” (Billen et al. 1991; Bouwman et al. 2013), but the effectiveness of the filter varies broadly with the physico-chemical attributes as well as the biological configuration of the ecosystem. Particularly, erosion of the filter function with hypoxia and loss of deep-burrowing benthic macrofauna has been shown (Conley et al. 2009; Norkko et al. 2012; Carstensen et al. 2014b). Whereas the individual biogeochemical processes underlying the coastal filter are generally well understood, the rates of these processes and the influence of various environmental factors on these rates are not well constrained (e.g. Ruttenberg 2003; Giblin et al. 2013). Due to the variable nature of the coastal environment, the dominant pathways can also change considerably and abruptly over time and space. Consequently, integrative biogeochemical models, based on established relationships of different pathways as a function of environmental factors, are needed to assess the coastal filter function.

The main objective of this review is to identify the major pathways for nutrients (N, P and Si) and organic matter, as well as their drivers, within the Baltic Sea coastal zone. The biogeochemical processes transforming, retaining and removing nutrients and organic matter have only been measured directly for a few selected coastal ecosystems, providing fragmented and limited knowledge to assess the coastal filter across the broad range of coastal ecosystems in the Baltic Sea. Here, we will synthesize the recent developments in our understanding of coastal biogeochemistry, and we will discuss consequences for coastal management.

## Coastal ecosystems of the Baltic Sea

**Fig. 1:**
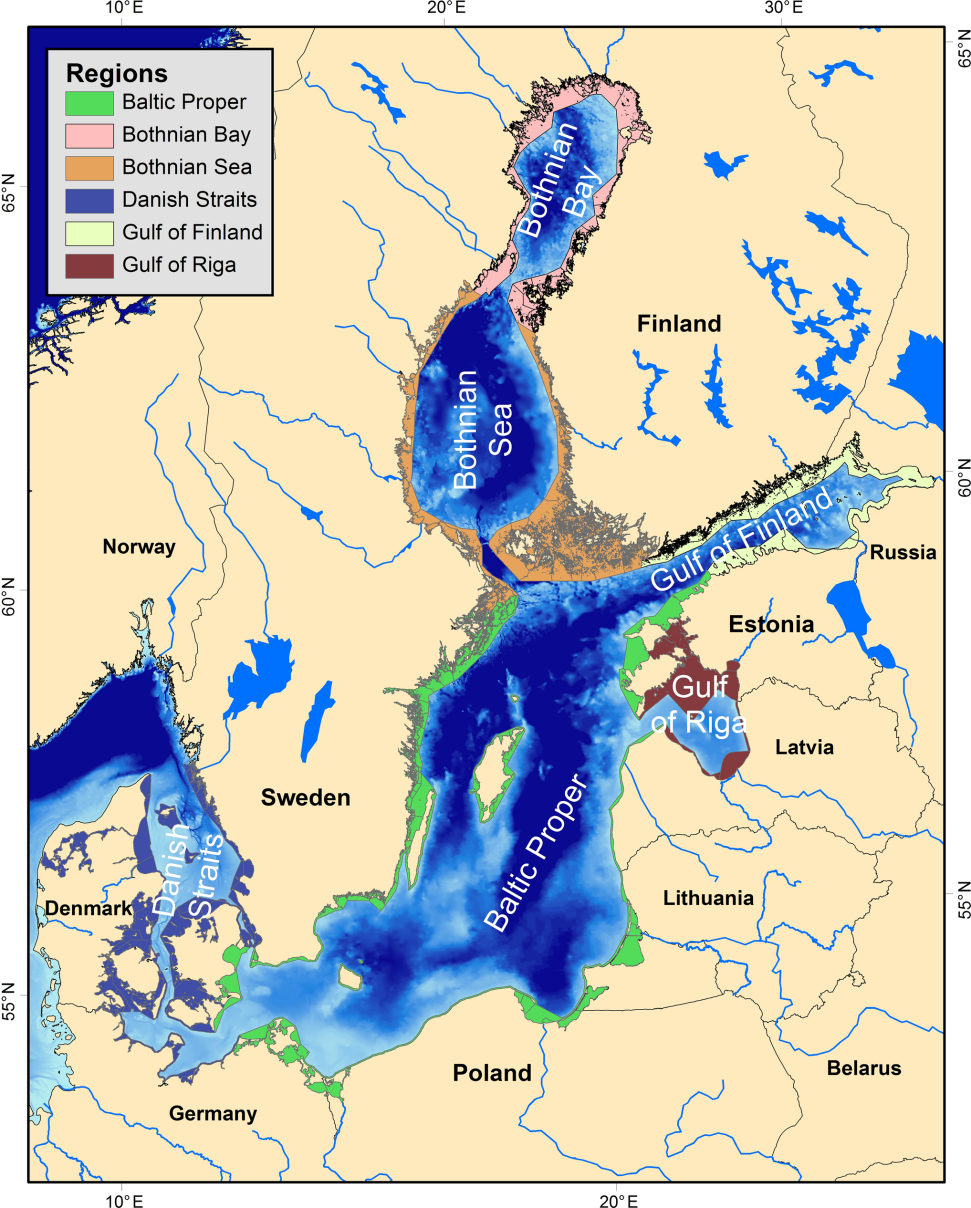
The Baltic Sea and its catchment with coastal ecosystems and their regions considered in the present study. Coastal ecosystems are delineated according to national definitions of water bodies according to the European Water Framework Directive

The Baltic Sea is a large tideless inland sea covering almost 400 000 km^2^ with a coastal periphery of about 8 000 km. However, due to the complex morphometry and fractal-like properties, it is difficult to quantify. The geological processes during the last glacial period and the Holocene have created diverse land- and seascapes around the Baltic Sea, changing from boreal archipelagos in the north, over long open coasts interrupted by lagoons in the southeast, to a drowned moraine landscape with many estuaries and embayments to the southwest. Consequently, coastal ecosystems exhibit large environmental and ecological gradients across the Baltic Sea from the entrance in the southwest to the three gulf extensions of the Baltic Proper to the north and east. Here, coastal systems in six regions are considered (Fig. 1).

**Fig. 2:**
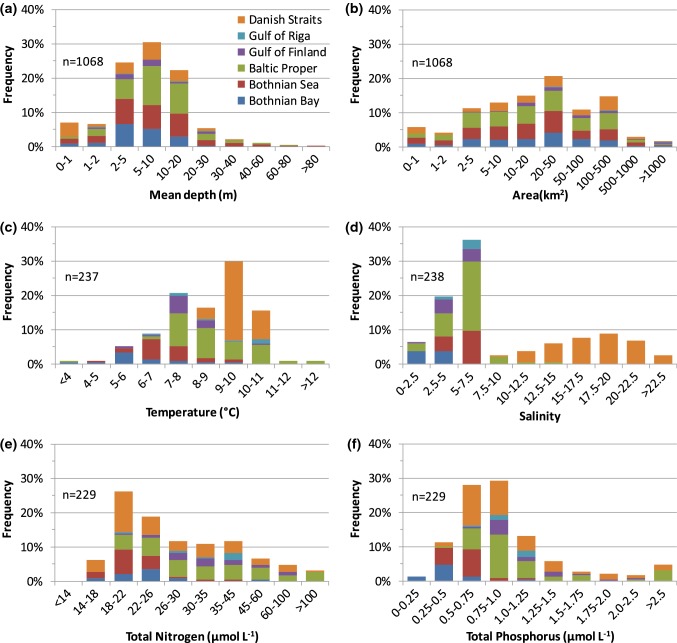
Distribution of mean depth (**a**), area (**b**), temperature (**c**), salinity (**d**), TN (**e**) and TP (**f**) across coastal systems in different regions of the Baltic Sea. Coastal systems were defined based on the national implementation of the European Water Framework Directive. The number of coastal systems used to characterize the distribution is inserted in each histogram. Mean temperature, salinity, TN and TP for the subsets of coastal systems were calculated from monitoring data (http://nest.su.se/bed/)

The majority of the coastal systems are shallow (< 20 m; Fig. 2a) with surface areas typically ranging from 2 to 500 km^2^ (Fig. 2b). The deepest systems are located in the Baltic Proper and Bothnian Sea, whereas the shallowest are found in the Danish Straits. All regions have a broad size span of coastal systems. There are marked latitudinal differences in both temperature and salinity. The annual mean temperature increases gradually from typically 5–6 °C in the Bothnian Bay to 9–10 °C in the Danish Straits (Fig. 2c). Similarly, mean salinity ranges from 1.5 to 4.9 in the Bothnian Bay with slightly higher ranges in the Bothnian Sea (2.8–6.5), Gulf of Finland (2.2–5.9), Gulf of Riga (4.7–6.0) and Baltic Proper (0.3–12.8), before a steep salinity increase occurs in the Danish Straits (9.8–25.8) (Fig. 2d). Microtidal currents are observed only in the Danish Straits, and wind conditions are generally more important for water exchanges between the coastal systems and the open waters.

Pronounced latitudinal differences in watershed properties are found as well. The catchment covers an area about 4.5 times larger than the sea itself, and the majority of freshwater input occurs through large rivers discharging into the Baltic Proper, the Bothnian Sea, the Bothnian Bay and the Gulfs of Finland and Riga (Mörth et al. 2007). In contrast, freshwater discharge to the Danish Straits occurs through many small and scattered streams. Moreover, there is a pronounced land-use gradient from dominantly forests in the north to agriculture in the south (Mörth et al. 2007). This gradient is also reflected in overall low nutrient concentrations in the Bothnian Sea and Bothnian Bay, whereas other regions exhibit a broader span in nutrient concentrations (Fig. 2e, f). In contrast, total organic carbon (TOC) inputs are high in the Bothnian Sea and Bothnian Bay due to dominance of peat soils in the catchments (Mattsson et al. 2005; Hoikkala et al. 2015). Another regional feature is the damming of rivers for hydropower, which is particularly pronounced in northern Sweden, trapping silica and potentially causing Si deficiency in coastal systems (Humborg et al. 2002).

Coastal hypoxia is a pronounced phenomenon in the Baltic Sea, due to stratification and low ventilation of bottom waters in combination with high inputs of nutrients and organic material (Conley et al. 2011). Many coastal systems are naturally prone to hypoxia due to the complex hydromorphology, even though inputs of allochthonous and autochthonous matter are low. Furthermore, hypoxia can be imported into coastal systems from the persistently hypoxic waters in the Baltic Proper (Carstensen et al. 2014a) or the seasonally hypoxic waters in the Danish Straits (Conley et al. 2007). Importantly, hypoxia significantly affects nutrient cycling, by altering biogeochemical processes, including those involving benthic organisms (Conley et al. 2007, 2009; Carstensen et al. 2014b; Gammal et al. 2017).

In addition to oxygen conditions, salinity is an important factor structuring the benthic community. As salinity decreases to the north and east, the number of marine species decreases, while the number of brackish water and limnic species increases (Remane 1934), creating an ecocline along which community composition successively changes (Attrill and Rundle 2002; Gogina et al. 2016). Many coastal systems experience salinities coinciding with the species diversity minimum described by Remane (1934). The benthic macrofauna community is generally rich in the Danish Straits (> 150 species), whereas the species diversity rapidly declines with salinity through the Baltic Proper towards the Bothnian Bay (Bonsdorff 2006; Villnäs and Norkko 2011; Törnroos et al. 2015). Similarly, low species diversity in the more brackish parts of the Baltic Sea is observed also for other organisms such as macroalgae and fish, whereas the number of aquatic vascular plant species increases from a few marine species in the southern Baltic Sea up to about 10 species of marine and limnic origin co-occurring in the northern Baltic Sea (Boström et al. 2014; Gustafsson and Norkko 2016).

The structure of biotic communities varies markedly in coastal zones of the Baltic Sea, dictated by coastal hydromorphology and associated environmental drivers. Even though the vascular plant diversity increases with decreasing salinity, the biomass of vascular plant communities decreases. For example, the biomass of eelgrass (*Zostera marina*) tends to be considerably higher in the high-salinity areas compared to the low-salinity areas (Boström et al. 2014). Regarding the biomass of benthic communities, high values are found in the Danish Straits as well as the Finnish archipelago (Gogina et al. 2016), where the number of benthic species is reduced but the functional diversity remains high (Törnroos et al. 2015). Indeed, the coastal zones of the relatively young Baltic Sea ecosystem are highly dynamic, creating a mosaic of habitats and numerous biotopes that host more diverse and abundant communities than open sea areas, and offer available niches for many non-indigenous species (Olenin and Leppäkoski 1999).

## Land–sea biogeochemical gradients

**Fig. 3:**
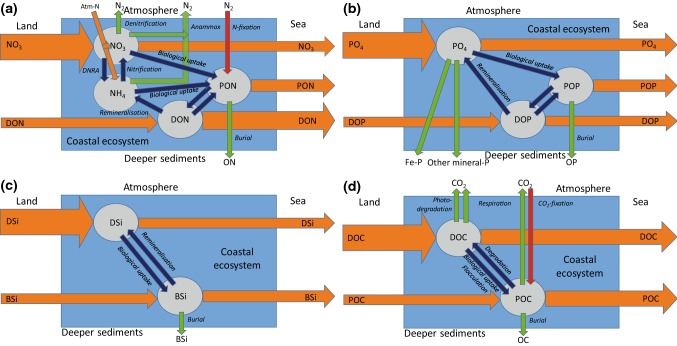
Major pathways for nitrogen (**a**), phosphorus (**b**), silica (**c**) and organic carbon (**d**) in the coastal zone. The coastal ecosystem box includes both the water column and the “biologically active” top sediment layer. Coastal stocks of different forms are shown as grey circles. Aquatic transports are shown in orange, removal processes in green, production processes in red and internal processes in dark blue. For the in–out net transport fluxes, the arrow sizes indicate relative magnitudes of the different components in a typical coastal ecosystem with significant inputs from land, whereas this is not the case for the arrows representing biological processes

In addition to the large regional differences across the Baltic Sea, gradients in salinity and concentrations of nutrients and organic matter from freshwater sources to the outer marine boundary are also present within the coastal ecosystems. Concurrently, coastal dynamics such as upwelling and resuspension add to the mixture of drivers that influence nutrient fluxes in the coastal zone (Heiskanen et al. 1998; Niemistö et al. 2018). These gradients shape biological communities with consequences for the biogeochemical processes, as nutrients and organic matter are transported from land to sea. The main biogeochemical processes responsible for the transformation, retention and removal of nutrients and organic matter in the coastal zone are illustrated in Fig. 3.

### Nitrogen processing

Nitrogen inputs from land enter the coastal zone mainly as dissolved inorganic nitrogen (DIN) and dissolved organic nitrogen (DON), and nitrate typically dominates input from watersheds with intense agricultural land use (Fig. 3a). Nutrient inputs are further augmented by atmospheric inputs, mainly deposited as nitrate and ammonia. On the other hand, nitrogen (N) typically leaves the coastal zone as organic nitrogen or dinitrogen gas following alterations in a complex network of processes that shift N between different inorganic and organic forms (Fig. 3a). However, the various N pathways are controlled by environmental factors such as availability of different nitrogen species and labile organic carbon, and redox conditions. For example, recycling processes such as dissimilatory nitrate reduction to ammonium (DNRA) may increase when oxygen is below a threshold of 100 µmol L^−1^, while denitrification becomes less pronounced (Jäntti and Hietanen 2012). Furthermore, N processing can be modulated by benthic communities (see below) and episodic events like upwelling and winter storms that are able to resuspend the upper sediment layer, including nutrients and microbial communities in the pore water (Happel et al. 2018).

Numerous studies on coastal processes highlight residence time as the major controlling factor for N processes (Middelburg and Nieuwenhuize 2000; Asmala et al. 2017). The longer water remains in the coastal zone, the more time microbial processes can be active to change concentrations of substrates and products. In addition, the coupling of water column processes with those in sediments is enhanced through deposition of particles from the water column. Lagoons and enclosed bays have the longest residence time because only small canals or inlets connect the water body with the open sea and the lack of tidal currents.

Denitrification, an important process removing N from the ecosystem, is regulated by oxygen concentrations and nitrate availability as well as temperature and labile dissolved organic carbon content in sediments and overlying waters (Piña-Ochoa and Álvarez-Cobelas 2006). This also holds for the Baltic Sea where such relationships have been confirmed in organic rich sediments (Hietanen and Kuparinen 2008; Deutsch et al. 2010). Denitrification in the oligotrophic coastal areas of the Bothnian Sea and Bothnian Bay is generally limited by availability of organic carbon in spring and nitrate in summer, and coupled nitrification–denitrification, fuelled by settling POM in deeper, stratified coastal areas appears to be the most important pathway for N removal (Hellemann et al. 2017). Thus, both sediment characteristics and phenology of organic matter sedimentation to the seafloor determine the activity of nitrogen removal processes in sediments.

Coastal zones also store nutrients as recalcitrant organic matter in sediments and as nutrients in pore waters. Depending on the type of sediments, the organic matter content can vary considerably but in most cases it is below 5% loss on ignition (LOI) when sediments are coarse grained (Thoms et al. 2018). These sediments have low storage of organic matter, and any input of organic matter is rapidly degraded to mineral nutrients, which may accumulate in the sediment or be released to the water column during wave action and resuspension (Precht and Huettel 2003). Interestingly, muddy sediments with high organic matter content (LOI of > 5–20%) show positive relationship with denitrification but lack high anammox rates (Jäntti et al. 2011). The reason may simply be that anammox bacteria are autotrophic and are thus outcompeted by denitrifiers as long as organic matter availability is high.

Nitrogen fixation can be an important additional N source for some coastal ecosystems and is typically carried out by diazotroph cyanobacteria such as the two characteristic taxa found in the Baltic Sea, *Aphanizomenon flosaque* and *Nodularia* spp. Approximately 50% of the fixed nitrogen is released within hours from the cyanobacteria as ammonia, supporting other microorganisms (Adam et al. 2016), such that the turnover time of ammonium is only 1 h (Klawonn et al. 2019). Cyanobacteria cells remain positively buoyant during their lifetime and thus contribute to seaward export (Vybernaite-Lubiene et al. 2017). However, cells sink at the end of their growth period, and senescent cells release most of their nitrogen to the water column as DON or DIN. A detailed seasonal nitrogen budget for the Curonian Lagoon, located along the Lithuanian and Kaliningrad coast, showed that only a minor fraction of cyanobacterial organic N was immediately remineralized to nitrate and then denitrified in the sediments (Zilius et al. 2018). Consequently, most of the N losses over the annual cycle occurred, not in late summer after the cyanobacteria bloom, but in winter. This finding is surprising since denitrification is temperature dependent, assuming that higher temperatures in summer would stimulate denitrification as observed in other regions of the Baltic Sea (Hietanen and Kuparinen 2008), but in the Curonian Lagoon denitrification in summer is nitrate limited and a thin oxic layer at the surface sediment (< 1 mm) significantly reduces nitrate production by nitrifiers (Zilius et al. 2018). Over the entire year, N removal by denitrification is approximately balanced by N fixation by cyanobacteria, although the two processes are temporally decoupled (Zilius et al. 2018).

**Fig. 4:**
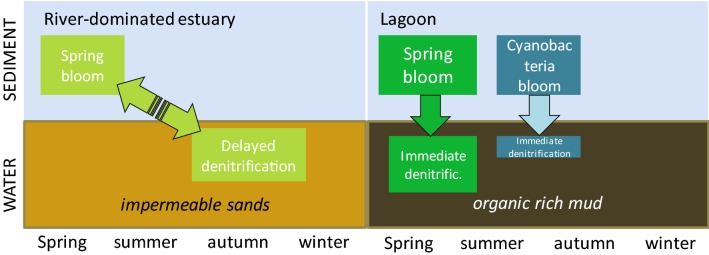
Conceptual diagram of seasonal differences in the denitrification pathway for the oligotrophic Öre River estuary and the eutrophic Curonian Lagoon. Denitrification in the Curonian Lagoon fuelled by the spring bloom is relatively more important than denitrification fuelled by the summer cyanobacteria bloom as indicated by the sizes of the boxes. Note that the sediment composition differs between the two sites

Comparing the Curonian Lagoon with the contrasting oligotrophic Öre River estuary in the Bothnian Bay, substantially seasonal differences in denitrification rates are observed, mainly driven by regional differences in temperature and inputs of N and organic matter (Fig. 4). In the Öre River estuary, denitrification was the largest in summer and fuelled by degradation of organic material from the spring bloom (Hellemann et al. 2017), whereas in the Curonian lagoon denitrification was large immediately after the spring bloom but low during the summer cyanobacteria bloom (Zilius et al. 2018). How organic matter production in surface waters fuels sediment removal process thus depends on a variety of site-specific interactions (Bartl et al. 2019).

### Phosphorus processing

Coastal systems act as key sites for transformation and removal of phosphorus (P) from land and from adjacent marine areas. As with other nutrients, phosphorus can cycle between dissolved and particulate forms many times in the coastal zone (Fig. 3b), with this recycling being strongly affected by interactions with other elemental cycles such as those of oxygen, iron (Fe), sulphur (S) and manganese (Mn) (e.g. Ruttenberg 2003; Slomp 2011). Three forms of solid-phase P are traditionally thought to account for most of the P removal in the coastal zone: (1) organic P, (2) Fe-oxide bound P and (3) authigenic apatite, which is a calcium phosphate mineral (Ruttenberg 2003). Most P removal takes place through burial in fine-grained sediments. Recent work, however, shows that in sediments with a low salinity, Mn-phosphates and vivianite, an Fe(II)–P mineral, may also form, with the latter acting as a major sink for P (Slomp 2011; Egger et al. 2015).

A recent compilation of total P burial rates for coastal areas in the Baltic Sea (Asmala et al. 2017) showed that burial rates of P are spatially highly variable, but generally increase from the open coast and coastal embayments (< 1 g P m^−2^ yr^−1^) to shallow lagoons, archipelagos and deeper estuaries (> 1 g P m^−2^ yr^−1^). The rate of sedimentation plays an important role in controlling P burial, with higher sedimentation rates being associated with greater P burial (Asmala et al. 2017).

For most coastal areas in the Baltic Sea, detailed studies of the permanent burial forms of P in the sediment are lacking. Nevertheless, the few available data suggest regional differences, with the burial forms depending mainly on organic matter loading, the availability of sulphate and iron input. Organic P is typically important as a P sink wherever fine-grained anoxic sediments accumulate relatively rapidly in a eutrophic environment. Examples of systems where this is the case include Aarhus Bay in Denmark (Jensen et al. 1995), the Gulf of Gdańsk in Poland (Lukawska-Matuszewska and Burska 2011), the Stockholm Archipelago in Sweden (Rydin et al. 2011), the Archipelago Sea in Finland (Lukkari et al. 2009) and estuaries in the Gulf of Finland (Lukkari et al. 2008). In these areas, apatite is often an important sediment component. However, the concentration of apatite only rarely increases with depth suggesting that, in many cases, its source is terrestrial and not authigenic (i.e. formed in situ; see Ruttenberg 2003). This particularly holds for the northern areas, such as the Gulf of Finland and the Archipelago Sea (Lukkari et al. 2008, 2009).

**Fig. 5:**
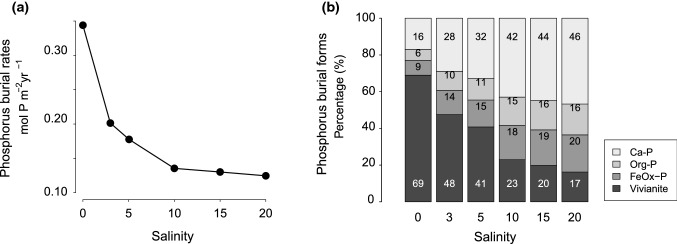
Results of a sensitivity analyses of a reactive transport model for a site in the coastal zone of the Bothnian Sea illustrating the role of salinity in controlling P burial in the region (Lenstra et al. 2018)

The Gulf of Bothnia is characterized by low salinity waters and inputs of organic carbon and iron from oligotrophic and often pristine rivers. A recent study in the Öre River estuary highlights that the high iron input allows for high rates of Fe-bound P burial in the coastal zone of the Bothnian Sea, with both Fe-oxides and vivianite acting as key sinks, vivianite formation being enhanced by low salinities (Fig. 5; Lenstra et al. 2018). This mechanism was reported earlier to be active in the deeper parts of the Bothnian Sea (Egger et al. 2015) and likely explains how this area of the Baltic Sea can act as an effective sink for P imported from the Baltic Proper (Savchuk 2005; Asmala et al. 2017).

### Silica processing

Coastal processes modulate the delivery of silica (Si) to adjacent marine areas (Billen et al. 1991). Si comes into estuaries from the production of weathering both as particulates and as dissolved Si (DSi), e.g. the reactive form taken up primarily by diatoms and converted into biogenic silica (BSi) (Fig. 3c). Substantial amounts of reactive BSi can also be carried by rivers (Conley 1997). Recent studies show that land use in watersheds, especially agricultural activity, amplifies the export of Si (Mangalaa et al. 2017).

Diatom growth, especially in long residence time systems such as lagoons, can substantially reduce DSi concentrations (e.g. Vybernaite-Lubiene et al. 2017). BSi sedimentation can be high in coastal areas, but much of the BSi that is sedimented can be regenerated and released back into the water column as DSi with the highest rates often observed in summer and lowest in early spring (Pastuszak et al. 2008; Tallberg et al. 2017). This internal Si recycling can sustain continued diatom blooms. Although there are a number of dated sediment cores with BSi accumulation, few estimates of Si retention are available for coastal ecosystems in the Baltic Sea; in the shallow Oder River estuary between Germany and Poland, only ca. 5% of the Si is retained in the estuary annually due to intense recycling (Pastuszak et al. 2008). Strong benthic–pelagic coupling occurs in the shallow water of Baltic coastal ecosystems (Griffiths et al. 2017). Benthic fauna, especially deep-burrowing chironomid larvae and polychaetes enhance DSi release from sediments (Benelli et al. 2018; Kauppi et al. 2018; Thoms et al. 2018). However, the presence of active benthic microalgae can reduce or reverse DSi efflux (Benelli et al. 2018).

The Baltic Sea is one of many aquatic systems globally that show long-term declines in DSi concentrations due to anthropogenic alteration of the biogeochemical Si cycle (Conley et al. 2008). River damming has lowered the fluxes of DSi to the Baltic Sea by ca. 45% (Humborg et al. 2006). Enhanced accumulation of BSi from eutrophication is also observed in sediments from the open waters of the Baltic Sea, but long-term trends in BSi accumulation from the coastal zone generally do not show increased BSi accumulation (Clarke et al. 2006; Ning et al. 2016).

### Organic matter in the coastal environment

Terrestrial inputs via rivers and streams are the dominant sources of organic matter (OM) to coastal areas of the Baltic Sea. The quantity and characteristics of these inputs of allochthonous OM are driven by conditions in the catchment, such as precipitation, land use and soil type (Whitehead et al. 2009; Autio et al. 2016). In general, allochthonous OM inputs to the Baltic Sea are characterized by relatively large molecules with pronounced humic-like properties and high bioavailability (Asmala et al. 2013), due to the short processing time from terrestrial sources to the coastal zone. However, autochthonous sources may exceed the contribution of allochthonous sources in eutrophic areas with high inorganic nutrient and low OM inputs. Part of the autochthonous production is released to the surrounding water continuously during the growth season as dissolved organic matter (DOM), and part of the production settles to the surface sediment as particulate organic matter (POM) (Fig. 3d). The cycling of these two distinct pools of OM is different, as the dissolved exudates support primarily heterotrophic bacteria (Attermeyer et al. 2018) and POM in the sediment surface is an essential food source for benthic heterotrophic communities. Further, over 90% of riverine OM input is in dissolved form (Mattsson et al. 2005), whereas POM from autochthonous production is relatively more important than allochthonous POM.

OM in the coastal environment is subjected to multiple processes transforming, retaining and removing it (Fig. 3d). One of the key abiotic mechanisms affecting OM quantity and characteristics is the exchange with the open sea (mixing), where concentrations are generally lower and characteristics differ from that of the terrestrial or autochthonous inputs. As mixing occurs simultaneously with transformation processes in coastal areas, the effects of these two are often difficult to delineate. In estuaries with relatively high freshwater input and short freshwater residence time, mixing alone can explain 70–80% of the observed variability in OM quantity and characteristics (Asmala et al. 2016). In addition to physical mixing, biotic and abiotic processes, including heterotrophic consumption, photochemical degradation and flocculation, shape the organic matter pool in coastal environments, and these processes often occur simultaneously or in repeated cycles along the aquatic continuum from land to sea (Xenopoulos et al. 2017).

In the coastal Baltic Sea, bacterial growth efficiency ranges between 0.25 and 0.41 (Asmala et al. 2013), which means that more than half of the consumed organic carbon is respired as CO_2_. Despite salinity being a defining factor for many biogeochemical processes in coastal environments, bacterial processing of DOM in coastal Baltic Sea is driven largely by OM quality rather than salinity (Kaartokallio et al. 2016). Bacterial utilization of OM is selective, as the more bioavailable organic fractions are utilized first (Hansell 2013). This is apparent for phytoplankton-derived fresh autochthonous DOM, which is rapidly (hours–days) transformed from labile to more recalcitrant DOM by the heterotrophic bacteria (Asmala et al. 2018).

In surface coastal waters, sunlight is absorbed by DOM and photodegradation can transform biologically recalcitrant OM into labile constituents and support bacterial growth (Moran and Zepp 1997). In general, terrestrially derived organic matter is more susceptible to photodegradation than autochthonous DOM (Zhu et al. 2017), and in this process, bulk characteristics of organic matter change from terrestrial to marine (Dalzell et al. 2009). In the Baltic Sea, photodegradation of DOM is estimated to produce the equivalent of 13–23% of the annual atmospheric deposition of inorganic N and to support annual bacterial uptake equivalent to 9–16% of the riverine total organic carbon inputs (Aarnos et al. 2012).

Flocculation is a process, where organic molecules aggregate into larger ones due to increase in ionic strength of the solution (Gregory and O’Melia 1989). In estuarine environments, the dissolved salts in seawater cause DOM in the river water to flocculate in a process that is both selective and nonlinear (Asmala et al. 2014). Large, humic-like molecules are more susceptible to flocculation than the bulk DOM, and also dissolved iron is effectively removed (Jilbert et al. 2018). Flocculation occurs throughout the salinity gradient, but it is most efficient already at salinities 1–2, where up to 16% of DOC is removed from the dissolved phase in Finnish estuaries (Asmala et al. 2014).

**Fig. 6:**
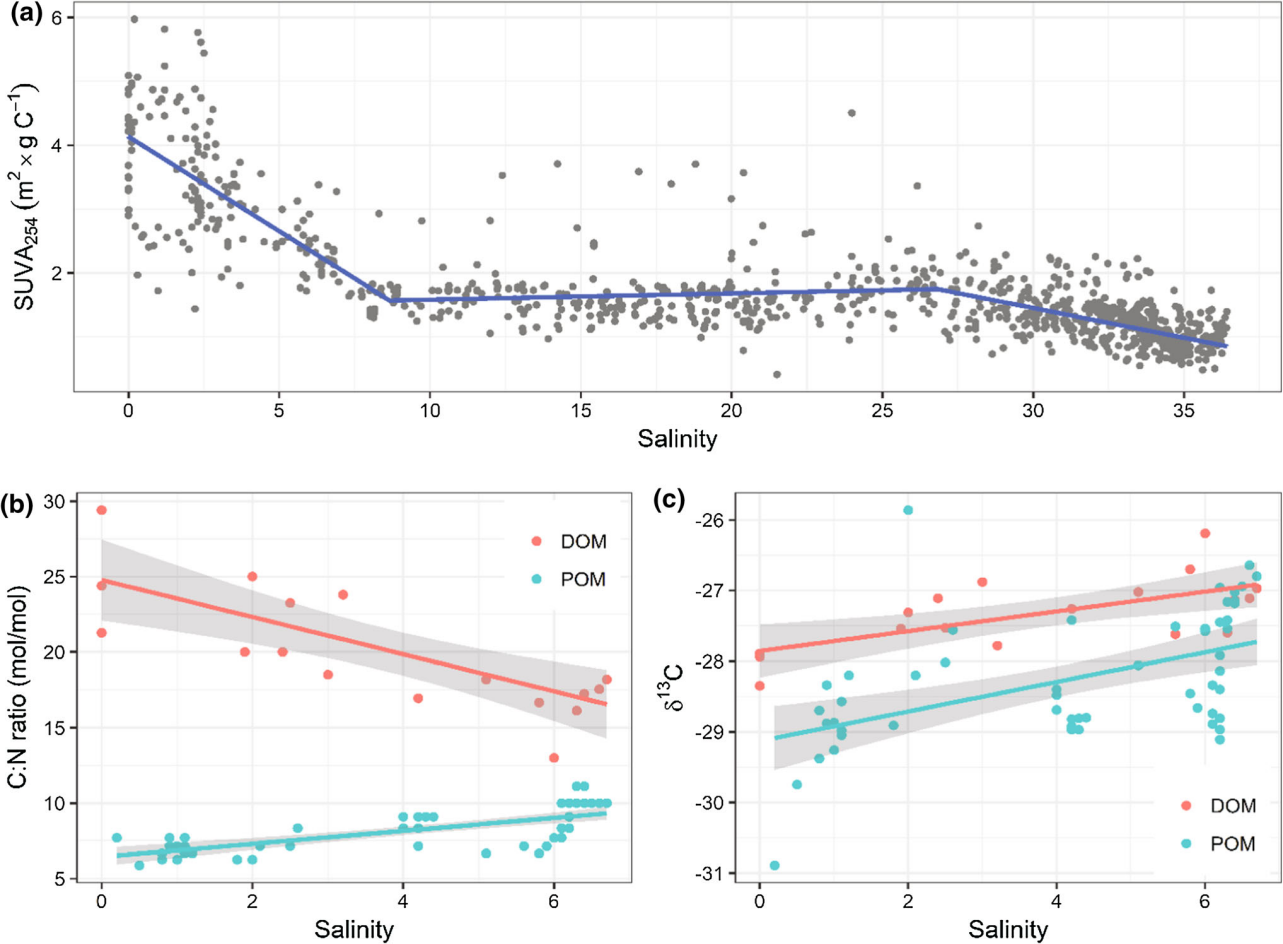
Changing organic matter characteristics along the salinity gradient: **a** DOC-specific UV absorbance (SUVA_254_), **b** C:N ratio and **c** stable isotope δ^13^C values in dissolved and particulate organic matter pools. Redrawn from Massicotte et al. (2017) (**a**), and Jilbert et al. 2018 (**b**, **c**)

AQThese processes altering the organic matter pool vary broadly in terms of their relative importance among coastal ecosystems, but some general patterns can be observed. During the transit through the coastal filter, DOM is bleached, i.e. the proportion of coloured DOM (CDOM) relative to dissolved organic carbon (DOC) concentration decreases as indicated by decreasing DOC-specific UV absorbance (SUVA; Weishaar et al. 2003) along the salinity gradient (Fig. 6a; Massicotte et al. 2017). This is caused mostly by photodegradation and flocculation, which both are processes that are selective to coloured and humic-like organic compounds. The C:N ratio decreases in the dissolved fraction and increases in particulates (Fig. 6b). The C:N ratio differs strongly between the two fractions in fresh water, but approaches a common ratio value during transition from land to sea. The relatively higher loss of carbon during the transit may be the result of bacterial processing of the DOM pool, as the continuous heterotrophic consumption releases a portion of the organic pool as carbon dioxide to the atmosphere, thus, reducing the terrestrial subsidies further down the salinity gradient. Isotopic signature of organic carbon also changes towards higher values (Fig. 6c), indicating an increasing contribution from planktonic sources to the organic matter pool.

## Biological effects on biogeochemistry

The biota function as key modulator of biogeochemical cycles within coastal ecosystems, where the close coupling between benthic and pelagic habitats enables dynamic exchanges of energy, carbon and nutrients through different pathways (Griffiths et al. 2017). By degrading, metabolizing and remineralizing OM, transforming and retaining nutrients, the biological components filter land-derived carbon and nutrients before they reach the open sea (McGlathery et al. 2007; Lloret and Marin 2011; Asmala et al. 2017). Primary producers, ranging from phytoplankton and microphytobenthos to macroalgae and vascular plants, form the foundation of food webs and are crucial for the energy transfer and nutrient filtering in coastal ecosystems. For instance, vascular plants such as seagrasses influence primary production and nutrient fluxes (Gustafsson and Norkko 2016; Staehr et al. 2018) as well as resuspension dynamics and sediment trapping in seascapes (Joensuu et al. 2018), while the sediment burial of macroalgae significantly contributes to the carbon sequestration in coastal environments (Krause-Jensen and Duarte 2016; Röhr et al. 2018). Secondary producers, such as benthic invertebrates, affect the cycling of carbon and nutrients directly through feeding on organic matter, production and excretion, and indirectly through bioturbation and bioirrigation (Benelli et al. 2018, 2019; Kauppi et al. 2018; Thoms et al. 2018; Janas et al. 2019). Benthic invertebrates can have a strong influence on sediment resuspension (Joensuu et al. 2018) and the retention and removal of carbon and nutrients by improving conditions for P-binding in the sediment and by facilitating N removal through nitrification-coupled denitrification (Moraes et al. 2008; Benelli et al. 2018). This affects benthic metabolism and nutrient exchange across the sediment–water interface (Thoms et al. 2018; Gammal et al. 2019).

The effects of biodiversity on ecosystem functioning are highly context dependent across scales and between locations (Snelgrove et al. 2014). Gustafsson and Norkko (2019) showed that the species composition of the vascular plant community changed along an exposure gradient in the Gulf of Finland, but the same key functional trait influencing primary production, plant height, remained the same. Furthermore, Gustafsson and Norkko (2016) demonstrated that inorganic N fluxes did not change in the presence of nine different vascular plant species but the plants enhanced the flux of dissolved organic nitrogen, while Angove et al. (2018) showed that the short-term nitrogen uptake dynamics of various plant species is largely driven by the presence and biological traits of the species.

Active sediment reworking by deep-burrowing benthic species is important at site-level scales. High abundance of bioturbators stimulates remineralization and nutrient effluxes at sandy coastal sites, even compared to sites with organically enriched muddy sediments and large reservoirs of dissolved nutrients, but a lower presence of the important bioturbators (Thoms et al. 2018). Indeed, Benelli et al. (2018) showed that the combined effect of bioturbating fauna (Chironomids) and microphytobenthos can strongly affect the internal nutrient cycling of a shallow coastal system, and even exert a bottom-up control of pelagic primary production. Still, the role of specific benthic species or functional traits for sediment nutrient recycling is highly complex, especially in heterogeneous coastal areas. For example, Gammal et al. (2019) explored spatial changes in solute fluxes over the sediment–water interface in a coastal area, and demonstrated that the benthic fauna (abundance and biomass) could account for 25% of the variability in solute fluxes, whereas environmental variables (e.g. temperature, roots, sediment organic matter and vegetation cover) accounted for 20%. The fauna played a large role in muddy sediments, while environmental drivers were more important in sandy sediments.

In addition to such spatial variability, seasonal variation in environmental factors affects nutrient remineralization both directly, and through effects on the biotic communities (Kauppi et al. 2017). Kauppi et al. (2017) reported high fluxes of oxygen and inorganic nutrients across the sediment–water interface during spring, largely explained (92%) by the dominant macrofaunal species in this study (the invasive spionid polychaete *Marenzelleria* spp. and the bivalve *Limecola balthica*). In contrast, during wintertime, the influence of *Marenzelleria* spp., even though abundant, on solute fluxes was negligible at the same coastal site. Still, this important invasive species can exert a great overall effect on ecosystem functioning, as it improves P retention through enhanced sediment oxygenation (Maximov et al. 2014, 2015; Isaev et al. 2017). It has also been noted to increase sediment nitrogen release, enhancing N:P ratios, which possibly could mitigate the production of cyanobacteria (Maximov et al. 2014, 2015).

**Fig. 7:**
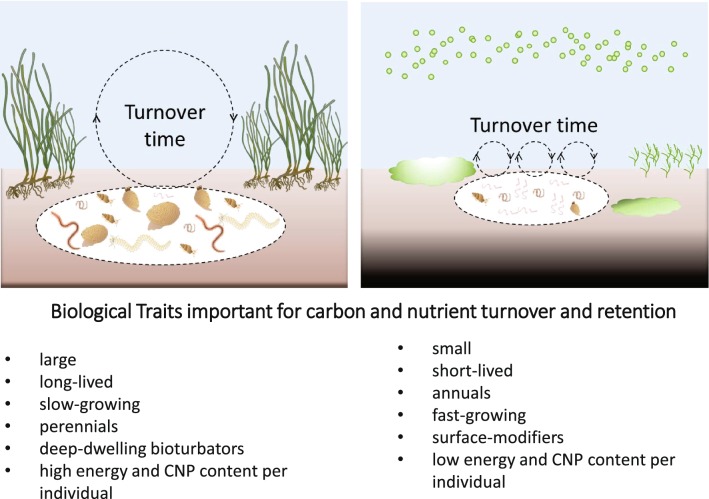
The effects of plants and animals on turnover time in the coastal filter, which are largely dependent on what biological traits the organisms express. Large and long-lived species are likely to promote the removal and/or retention of carbon and nutrients (left). In contrast, species characterized as small and short lived will enhance the turnover rates of carbon and nutrients within the coastal zone (right). Increasing eutrophication can result in such a shift with major implications for the coastal nutrient filter. Images by Tracey Saxby, Dieter Tracey, Jane Thomas, Integration and Application Network, University of Maryland Center for Environmental Science (https://ian.umces.edu/imagelibrary/)

Although the roles of primary and secondary producers for the turnover of nutrients in the coastal filter vary substantially, their impacts can partly be evaluated by studying biological traits (Fig. 7). For example, nutrient turnover rates will depend upon whether the dominating primary producers are short-lived annuals such as cyanobacteria and filamentous algae that grow and take up nutrients rapidly or longer-lived perennials such as vascular plants and macroalgae that grow slower but also store and retain nutrients on longer time-scales compared to annual plants. This further suggests that the role of vegetation for nutrient filtering in the coastal zone can vary extensively depending on the local environmental gradients, dominating species communities and biological trait composition of the occurring species. Benthic communities in inner coastal areas, more prone to eutrophication, express biological traits (short life span, high metabolism and elemental content per individual) that enhance turnover rates of carbon and nutrients (Villnäs et al. 2019). In contrast, outer coastal areas, which are deeper and less-nutrient enriched, are dominated by large, long-lived and deep-burrowing species, likely to have a more prominent role for nutrient retention and removal from the coastal filter (Fig. 7). The impacts of long-term organic stress resulting from local nutrient over-enrichment on the benthic fauna is further manifested throughout the entire benthic food web, increasing the vulnerability and reducing the resilience of the coastal system over time and space (Nordström and Bonsdorff 2017). This underlines the importance of preserving healthy biotic communities, with biodiversity that can sustain an efficient filtering function in the coastal zone.

## Management of the coastal zone

Our review demonstrates that the potential of coastal ecosystems to attenuate nutrients depends on the physical environment (topography, sediment type, residence time) as well as on the characteristics of the biological communities. Obviously, these fundamental ecological and biological entities, processes and functions need to be considered in evaluating management options for these coastal ecosystems. However, the main challenge is that different coastal types display varying capacities to capture and attenuate nutrients via sediment storage or atmospheric losses, in addition to their complex interaction with the open sea. Thus, a simple uniform managerial framework based on measurements alone is inadequate, but rather modelling of these complex environments is needed in order to test and predict the outcome of various management options. However, coastal ecosystems are affected by local land-based inputs as well as conditions in the open waters and, therefore, coastal management needs to consider these larger scales. Indeed, there are coastal ecosystems that cannot achieve good ecological status from managing local nutrient inputs alone. The HELCOM Baltic Sea Action Plan (BSAP) covers the open waters and is an ambitious plan for improving the Baltic Sea environment with specific focus on eutrophication. However, harmonization with coastal management and policies like the Water Framework Directive (WFD) are not thoroughly considered in the BSAP. In addition, the effects of the coastal filter are only partly considered and coastal management is not explicitly incorporated in the BSAP.

The complexity of the biogeochemical processes and biotic interactions implies that responses to nutrient management are nonlinear and involve substantial time lags. While the filtering capacity is not necessarily constant, it could be managed to improve the nutrient processing capacity of coastal ecosystems and reduce nutrient inputs to the open waters. However, it is difficult to assess the filtering capacity, as coastal responses to management can take many years before a new balance in nutrient content is obtained (Almroth-Rosell et al. 2016). A nutrient-reduction scenario in the Stockholm Archipelago showed a relatively fast response in the filter efficiency of N, whereas the filter efficiency of P first decreased, associated with the N response, before reaching a higher efficiency after 18 years (Almroth-Rosell et al. 2016).

A major fraction of nutrient inputs to the Baltic Sea passes through large estuarine systems such as the Curonian, Oder and Vistula lagoons, where 14–88% of total N and 27–89% of total P inputs are retained or removed (Grelowski et al. 2000; Vybernaite-Lubiene et al. 2017). Some coastal areas around Sweden remove more than 100% of the land inputs and hence have a filtering function on the open Baltic Sea water (Edman et al. 2018). Edman et al. (2018) estimated that 54% and 70% of total N and total P inputs from Sweden were retained in the coastal zone, values somewhat higher than those estimated by Asmala et al. (2017) for the entire Baltic Sea coastal zone (16% and 53%, respectively), who also suggest greater nutrient efficiency in the Swedish coastal zone, particularly for phosphorus.. However, Savchuk (2018) questioned this high filter efficiency for phosphorus in the coastal zone in the context of the overall nutrient budget of the Baltic Sea. Asmala et al. (2017) estimated that each year the entire Baltic coastal zone removes more than 100 000 tonnes N by denitrification and more than 16 000 tonnes P is permanently buried in the sediments, but these numbers cover large regional differences as well as large variation among coastal systems (Edman et al., 2018).

Both Edman et al. (2018) and Asmala et al. (2017) find that archipelagos and coastal lagoons are more efficient nutrient filters than other coastal types, but both studies also report large spread in removal rates and filter efficiency. Edman et al. (2018) suggest that the filter capacity primarily depends on physical characteristics, such as overturning times and mean water depth. For the Stockholm Archipelago, Almroth-Rosell et al. (2016) concluded that the oxygen levels in the bottom water are of great importance for the filter efficiency, and the presence of oxygen is a precursor for sediment-dwelling organisms enhancing nutrient removal rates (Norkko et al. 2012). Thus, coastal areas such as the inner Stockholm Archipelago can maintain high filter efficiency as long as oxygen conditions remain good. The inner Stockholm Archipelago is a prominent example of nutrient management improving the efficiency of the nutrient filter. The Baltic Sea has experienced phases of both eutrophication and oligotrophication during the last 50–100 years (Reusch et al. 2018), and the experiences of nutrient management could be useful in other areas where nutrient reductions will be needed to mitigate the adverse effects of eutrophication (e.g. the expansion of hypoxia in the East China Sea and the Gulf of Mexico).

## Conclusions

The Baltic Sea harbours a diverse set of coastal ecosystems, exhibiting broad ranges in physical setting, nutrient status and biological configuration. Consequently, the coastal filter functioning is similarly diverse, ranging from pure transport to efficient nutrient removal, even with coastal systems trapping phosphorus from the open Baltic Sea. Denitrification rates are high in lagoons receiving high inputs of nitrate and organic material, but low N:P ratios can promote N-fixing cyanobacteria, lowering the filter function, thus underlining the importance of managing inputs of both nitrogen and phosphorus. Seasonal variations in timing of organic matter sedimentation, nitrate availability and redox conditions are important controls for the N pathways, with variable removal efficiency among coastal ecosystems. Archipelagos are important for trapping phosphorus with burial strongly coupled to sedimentation rates, but the forms of buried P and their stability vary considerably across the Baltic coastal zone, mainly in response to salinity. Less is known about the burial of biogenic silica, although indications are that Si-removal is considerably lower than N and P removal. Organic matter undergoes large changes in both quantity and quality along the coastal gradient and these changes are tightly linked to the nitrogen and phosphorus cycles. The presence of specific biological traits can significantly enhance the coastal filter function and consequently, it is paramount to manage the coastal ecosystem to maintain the biological integrity and optimize the removal of nutrients and organic matter.
